# Postpartum bonding and association with depressive symptoms and prenatal attachment in women with fear of birth

**DOI:** 10.1186/s12884-021-04367-3

**Published:** 2022-01-25

**Authors:** Ingegerd Hildingsson, Christine Rubertsson

**Affiliations:** 1 Department of Women’s and Children’s Health, Uppsala University, Uppsala, Sweden; 2 Department of Health Science, Lund University, Lund, Sweden

**Keywords:** Depressive symptoms, Fear of birth, Pregnancy, Prenatal attachment, Postpartum bonding, EPDS

## Abstract

**Background:**

Co-morbidity is prevalent in women with fear of birth. Depressive symptoms and lack of prenatal attachment might influence the postpartum bonding between the mother and the new-born.

**Aim:**

To examine the underlying dimensions of the Postpartum Bonding Questionnaire and to investigate associations between depressive symptoms, prenatal attachment and postpartum bonding in women with fear of birth.

**Methods:**

A longitudinal study comprising 172 women with fear of birth. Data were collected by questionnaires in mid- and late pregnancy and two months after birth. The Edinburgh Postnatal Depressive Scale, Prenatal Attachment Inventory and Postpartum Bonding Questionnaire were investigated.

**Results:**

Two factors of the Postpartum Bonding Questionnaire were identified: Factor 1 mirrored caring activities and the women’s perceptions of motherhood, whereas Factor 2 reflected negative feelings towards the baby. The Postpartum Bonding Questionnaire was negatively correlated with the Prenatal Attachment Inventory and positively with The Edinburgh Postnatal Depressive Scale.

Women with fear of birth and depressive symptoms both during pregnancy and postpartum showed the highest risk of impaired bonding after birth. Primiparity and being single were also associated with impaired bonding.

**Conclusion:**

A focus on women’s mental health during pregnancy is necessary in order to avoid the negative effects of impaired bonding on the infant. Depressive symptoms could be concurrent with fear of birth and, therefore, it is important to determine both fear of birth and depressive symptoms in screening procedures during pregnancy. Caregivers who meet women during pregnancy need to acknowledge prenatal attachment and thereby influence adaptation to motherhood.

## Background

Mother–to-infant bonding is a process than can start during pregnancy and develops after birth [1]. Adaptation to motherhood is closely linked with the development of an emotional bond with the baby, and physical and mental health problems have been shown to be important indicators of impaired bonding [2]. Women’s mental health during the childbearing period is of growing focus mainly due to the prevalence of mental health problems [3] and the effect that such problems might have on the child [4].

Maternal-infant bonding refers to the process characterised by parental feelings and emotions towards the infant and the connection developed with the child [5, 6]. The process and relation between the mother and the baby starts during pregnancy, and is referred to as prenatal attachment [7]. Muller and Mercer described prenatal attachment as ‘the unique relationship between the mother and her unborn baby’ [7]. This connection implies communication with the unborn baby, fantasies and planning for the future activities with the baby [8].

A recent systematic review of 19 scientific studies showed that the majority of studies indicated positive relationships between prenatal attachment and later bonding [9]. Three of the included studies in the review reported a negative association between prenatal attachment and impaired bonding after birth [10–12]. Depressive symptoms during pregnancy are quite common and affect approximately 7–16% of pregnant women [13, 14]. Depressive symptoms after birth are present in 9–12%, and around 12% of women show depressive symptoms during the overall perinatal period [15]. Depressive symptoms are associated with lower prenatal attachment as shown in a population-based study of 718 pregnant women [16]. Alhusen reported in a review from 2012 [17] that pregnant women with mental health problems, such as anxiety and depression were more likely to present impaired maternal–fetal bonding. Reck et al. [18] also concluded that depression can negatively influence the interactions between the mother and the infant [18].

Fear of birth is also common in pregnant women and affects approximately 14% worldwide, as shown in a systematic review and meta-analysis based on 29 scientific papers [19]. Fear of birth has been linked to depressive symptoms as shown in another systematic review of 21 scientific papers, [20] and comorbidity between fear of birth and mental health problems are well known [21–23]. Co-morbidity refers to people who have a disease or condition also have one or more other diseases or conditions (https://dictionary.cambridge.org/dictionary/english/comorbidity).

Such fear can adversely affect a woman’s ability to build a relationship to the unborn [24] and the newborn baby [25]. Hence, comorbidity is prevalent in women with fear of birth. Pregnant women’s mental health needs to be further addressed, as it could affect the health of both the mother and the baby [26], the prenatal attachment [16], the postnatal bonding and parenting [18, 25], and children’s emotional health up to five years of age [4].

### Problem area

Previous studies have shown that depressive symptoms could adversely affect the development of the child and is associated with prenatal attachment but the link between emotional well-being, prenatal attachment and postnatal bonding in women with fear of birth is not fully investigated. Hence, the aim of the present study was to examine the underlying dimensions' of the Postpartum Bonding Questionnaire and to investigate associations between depressive symptoms, prenatal attachment and postpartum bonding in women with fear of birth.

## Methods

### Design

A longitudinal cohort study of a combined sample of pregnant women participating in treatment for fear of birth. One sample came from a randomized controlled trial (RCT) [27] and one sample from an experimental study where women were offered continuity with a known midwife [28].

### Setting

The randomized controlled trial was performed in one university hospital and two referral hospitals in Sweden and the experimental study in three referral hospitals. All women reported severe fear of birth. They were enrolled in antenatal care in their community, following an established national program. with a local midwife as the main caregiver.

### Participants and Procedures

Women in the randomized controlled trial were allocated to counselling with midwives or internet-based cognitive therapy [27]. The primary outcome for the RCT were levels of fear two months and one year after birth [29]. The women filled out a screening questionnaire when they came in for a routine ultrasound examination in gestational weeks 17–19. The screening questionnaire included the Fear Of Birth Scale (FOBS), [30] which was the basis for further recruitment to the study. The FOBS includes a question (‘How do you feel right now about the approaching birth?’). Women answered with an “X” on the two 100 mm response lines with the anchor words ‘Calm’- ‘Worried’ and ‘No fear’- ‘Strong fear’. Women reported to what extent they felt fear when thinking about the upcoming birth. The lines were measured separately, and subsequently added and averaged. A cut-off point of 60 or more indicated severe fear of birth [30]. Women who scored 60 or more on the FOBS were contacted by a research midwife and informed about the study. If they consented to participate, they received login details to the internet platform (www.u-care.se). After completing the first questionnaire, women were randomised to counselling with midwives or to internet-based cognitive therapy. All questionnaires were completed online. Details of the process are presented elsewhere [28]. A total of 258 women were included in the randomized controlled trial, but only those who completed all three questionnaires were included in the present study (n = 104). 

Women included in the experimental study (Auroraplus) consisted of 68 of the original 77 women who were offered counselling with midwives; and in addition, when possible, the counselling midwife provided birth assistance and intrapartum care. Women in the experimental study were referred to the counselling team by the antenatal midwife after FOBS screening or when disclosed fear of birth. They received oral and written information about the study, and if they agreed to participate, questionnaires were sent to their home address [28]. Continuity of care with a known midwife were given randomly, however, there was no midwife on call for births [31]. The inclusion in the present study implied that all three questionnaires were completed.

### Data collection

Data was collected by three questionnaires, the first in mid-pregnancy (gw 25), the second in late pregnancy (gw 36) and the third two months after birth. The questionnaires were similar regardless of given treatment. Background variables were collected in the first questionnaire completed in mid-pregnancy (age, marital status, level of education, country of birth, history of mental health problems, and parity).

**Depressive symptoms **were investigated in mid-pregnancy and two months after birth using the ten-item Edinburgh Postnatal Depression Scale (EPDS) [32], that has been validated in Sweden [33]. EPDS was analysed as a continuous variable and also dichotomised. A cut-off of 13 or above was used during pregnancy, [33] and a cut-off of 12 or above was used after birth [32] to classify women with or without depressive symptoms. To study comorbidity, a composite variable was thereafter created, based on the following dichotomised variables of EPDS: scores of 0 = No depressive symptoms during pregnancy or after birth; 1 = depressive symptoms during pregnancy but not after birth; 2 = depressive symptoms after birth but not during pregnancy; and finally, 3 = depressive symptoms on both occasions.

In late pregnancy prenatal attachment was assessed using the revised version of the Prenatal Assessment Inventory (PAI-R) [34]. PAI-R consists of 18 items divided into the three subscales: anticipation, differentiation and interaction. The 18 items were responded to on a Likert scale with 4-point responses (1 = almost never, 2 = sometimes, 3 = often, 4 = almost always). Higher scores indicating higher levels of prenatal attachment. The three subscales of PAI-R reflect fantasies and future plans for the baby (anticipation), the woman’s feeling for the baby and sharing the experiences with other people (interaction) and the woman’s knowledge about the baby’s personality and activities (differentiation).

The **Postpartum Bonding Questionnaire** (PBQ) was originally developed by Brockington et al. [35] and consists of 25 items, with four factors. The first factor was labelled *general emotional factor*, the second *anger towards and rejection of baby*; the third *infant-focus anxiety;* and the fourth, *risk of abuse*. During past years, PBQ has undergone several revisions, reflecting diverse factors or dimensions of bonding [18, 36–38], which usually resulted in a reduced number of items and failure to replicate the original version. In the present study we used the 16-item version of PBQ [18], where participants rate their agreement of the statements on Likert scales ranging from 0 (always) to 5 (never). The reason for using the 16-item version was mainly to fit the Swedish context, where certain items in the 25-item version would have yield less variability, especially items reflecting abuse if children, which is forbidden by law. Positively worded items were reversed, and higher scores indicate more severe and impaired bonding problems.

### Analysis

Descriptive statistics were used to present the background characteristics. The 16 items of the Postpartum Bonding Questionnaire [18] were subjected to a Principal Component Analysis (PCA) [39]. Prior to performing the PCA, the suitability of data was assessed by a Kaiser–Meyer–Olkin (KMO) measure of sampling adequacy and Bartletts’ test of sphericity. Only items loading 0.40 or more were included in the analysis. Three decision rules were used to identify the number of factors to retain: Kaiser’s criterion, retaining eigenvalues above 1; Cattell’s scree plot; and Horn’s parallel analysis where eigenvalues obtained from the PCA were compared with those from a randomly generated data file of the same size [39]. Only those factors with eigenvalues above the value obtained from the corresponding random data file were retained. Several analyses were obtained with 2–5 factors until the best model was achieved. Pearson’s correlation analysis was conducted between the instruments EPDS, PAI and PBQ. Mean scores and standard deviations were calculated between the factors identified in the PCA and women’s background characteristics.

## Results

The 172 participating women with fear of birth who underwent treatment during pregnancy formed the sample (Table 1). Most women were 25–35 years old (mean 31.07, SD 4.97, range 17–44 years), living with a partner (96.5%), born in Sweden (91.3%), and had a high level of education (65.7%). The majority had previous children (52.9%). Nearly one in three women (29.1%) presented with depressive symptoms during pregnancy as did 19% two months after birth. Two months after birth 5.2% of those without fear in mid pregnancy scored 12 or more on the EPDS, and 23 women (13.7%) scored high both during and after pregnancy. A total of 112 women (66.7%) had no sign of depressive symptoms at any time and 24 women in mid pregnancy (14.3%).

**Table 1:** Background of the participants

Women in the project	*n* = 172 *n* (%)
**Age groups**	
< 25 years	10 (5.8)
25–35 years	120 (70.2)
> 35 years	41 (24.0)
**Marital status**	
Living with a partner	166 (96.5)
Not living with a partner	6 (3.5)
**Country of birth**	
Sweden	157 (91.3)
Other country	15 (8.7)
**Level of education**	
High school or lower	59 (34.3)
University education	113 (65.7)
**Parity**	
Primiparas	81 (47.1)
Multiparas	91 (52.9)
**Depressive symptoms in mid pregnancy**	
EPDS < 13	122 (70.9)
EPDS 13 or more	50 (29.1)
**Depressive symptoms 2 months after birth**	
EPDS < 12	136 (81.0)
EPDS 12 or more	32 (19.0)

### Principal component analysis and inter-correlations among scales

The Kaiser–Meyer–Olkin (KMO) measure of sampling adequacy was 0.83, exceeding the recommended value of 0.60, and Bartlett’s test of sphericity was statistically significant (p 0.000), suggesting factorability of the data. Initially the PCA revealed five components with eigenvalues > 1, explaining 35.1%, 9.9%, 7.9, 6.7% and 6.5% of the total variance of 65.4%. The parallel analysis, however, only supported the retention of two factors for further investigation when compared to a randomly generated data matrix of the same size (16 variables, 172 respondents).

The two-factor solution explained 45.14% of the total variance, with Factor 1, 35.19% and Factor 2, 9.94%. To aid the interpretation of the two components, Oblimin rotation of the two-factor solution showed a clear pattern, with the all items loading on only one factor (see Table 2). The correlations among the factors were quite low (0.36), suggesting that the factors should not be combined to form a total score. However, in this solution, one item (My baby is difficult to comfort) was rejected due to lack of loading (< 0.40). The 16-item scale had an internal consistency of 0.86. The Cronbach alpha value for Factor 1 was 0.82 and for Factor 2, 0.75. Factor 1 included caring activities and the woman’s perceptions of motherhood, whereas Factor 2 involved negative feelings towards the baby. The two factors identified using the Principal Component Analysis were further investigated in relation to women’s sociodemographic background, their prenatal attachment and depressive symptoms. The pattern matrix and structure matrix of the items included in the factors are shown in Table 2.

**Table 2:** Result of the principal component analysis of the postpartum bonding questionnaire

	Pattern matrix		Structure matrix	
*n* = 172	**Factor 1**	**Factor 2**	**Factor 1**	**Factor 2**
**Items**				
I love my baby to bits (Rev)	**0.828**	0.094	**0.794**	0.205
I feel close to my baby (Rev)	**0.738**	-0.043	**0.754**	0.310
I love to cuddle my baby (Rev)	**0.663**	-0.117	**0.706**	0.357
My baby is the most beautiful baby of the world (Rev)	**0.646**	-0.036	**0.658**	0.269
I wish the old days when I had no baby would come back	**0.592**	-0.113	**0.633**	0.327
I feel happy when my baby smiles or laughs (Rev)	**0.557**	0.350	**0.613**	**0.518**
I feel trapped as a mother	**0.516**	-0.245	**0.605**	**0.432**
I feel distant from my baby	**0.489**	-0.341	**0.572**	**0.462**
I enjoy playing with my baby (Rev)	**0.466**	-0.293	**0.505**	0.283
I resent my baby	**0.463**	-0.116	**0.431**	0.148
My baby annoys me	0.092	**0.790**	0.377	**0.823**
My baby irritates me	0.078	**0.786**	0.363	**0.815**
I feel angry with my baby	-0.117	**0.731**	0.147	**0.689**
My baby winds me up	0.324	**0.554**	**0.521**	**0.661**
My baby makes me anxious	0.128	**0.479**	0.301	**0.525**
My baby is easily comforted	0.206	-0.255	0.299	-0.330
**Cronbach alpha value**	**0.824**	**0.752**		

Bold values indicate major loadings

The inter-correlations of the scales are presented in Table 3. Both factors on the PBQ were negatively associated with the subscales on Prenatal Attachment Inventory (PAI), with the exception of Factor 2, which was not significantly associated with the subscale of differentiation (Table 3). High levels of bonding impairment also showed higher summed scores of EPDS.

**Table 3:** Intercorrelations between factors obtained from the Postpartum Bonding Questionnaire, Prenatal Attachment Inventory, and Edinburgh Postnatal Depression Scale

	**Factor 1 PBQ**^**1**^	**Factor 2 PBQ**^**2**^
**Factor 2 PBQ (*n* = 172**)	0.584***	
**Prenatal attachment (PAI) (*n* = 150)**		
Anticipation (mean 16.30, SD 3.73) Range 8–24	-0.277**	-0.244**
Interaction (mean 16.95, SD 3.62) Range 8–24	-0.280**	-0.199*
Differentiation (mean 17.04, SD 4.45) Range 6–24	-0.235**	-0.072
**Depressive symptoms (*n* = 171)**		
EPDS^3^ in mid pregnancy (mean 9.64, SD 5.60) Range 0–24	0.259**	0.175*
EPDS postpartum (mean 7.53, SD 5.12) Ranges 0–27	0.566***	0.452***

^1^Factor 1 includes caring activities and the woman’s perceptions of motherhood

^2^Factor 2 includes negative feelings towards the baby

^3^*EPDS* refers to Edinburgh Postnatal Depressive Scale

### Background characteristics in relation to the factors

In Table 4 the mean scores of the two factors are presented in relation to women’s background characteristics. There were no differences in age, country of birth, level of education or previous mental health problems. Women who were not living with a partner in mid-pregnancy were more likely to score higher on both factors (Factor 1 caring activities and the woman’s perceptions of motherhood, and Factor 2 negative feelings towards the baby). This indicated that these women had more impaired bonding and negative feelings towards the baby. First-time mothers were also at higher risk of less favourable bonding in terms of having more negative feelings towards the baby.

**Table 4:** Mean scores (*SD*) of the factors of Postpartum Bonding Questionnaire in relation to background variables

	**Factor 1**	**Factor 2**
**Age groups**		
16–25 years (*n* = 10)	5.00 (4.13)	4.10 (1.72)
25–35 years (*n* = 120)	4,15 (5.22)	4.32 (3.41)
35 years or more (*n* = 40)	4.27 (3.94)	3.87 (3.25)
*p*-value	0.871	0.754
**Civil status**		
Living with a partner (*n* = 166)	4.03 (4.59)	4.07 (3.14)
Not living with a partner (*n* = 5)	10.20 (11.90)	7.80 (6.18)
*p*-value	0.005	0.012
**Country of birth**		
Sweden (*n* = 156)	4.36 (4.97)	4.25 (3.36)
Other country (*n* = 15)	2.60 (3.55)	3.46 (2.61)
*p*-value	0.079	0.295
**Level of education**		
High school or lower (*n* = 112)	4.08 (5.31)	3.62 (3.03)
University education (*n* = 59)	4.27 (4.64)	4.47 (3.41)
*p*-value	0.807	0.112
**Parity**		
Primiparas (*n* = 81)	4.56 (5.75)	4.97 (3.53)
Multiparas (*n* = 90)	3.88 (3.91)	3.46 (2.91)
*p*-value	0.364	0.003
**Previous mental health problems**		
No (*n* = 83)	4.07 (3.79)	4.19 (3.00)
Yes (*n* = 88)	4.34 (5.71)	4.17 (3.57)
*p*-value	0.720	0.965
**Depressive symptoms mid pregnancy**		
EPDS^1^ < 13 (*n* = 122)	3.48 (3.36)	3.86 (3.03)
EPDS 13 or more (*n* = 49)	6.02 (7.12)	4.95 (3.81)
*p*-value	0.002	0.051
**Depressive symptoms 2 months after birth**		
EPDS^1^ < 12 (*n* = 136)	3.31 (3.28)	3.61 (2.88)
EPDS 12 or more (*n* = 32)	7.75 (8.06)	6.65 (3.96)
*p*-value	0.000	0.000
**Trajectories of depressive symptoms**		
No depressive symptoms at any time	3.35 (3.29)	3.63 (2.87)
Depressive symptoms only in mid pregnancy	3.12 (3.27)	3.50 (2.99)
Depressive symptoms only postpartum	5.00 (4.18)	6.66 (3.90)
Depressive symptoms in pregnancy and	8.82 (9.00)	6.65 (4.07)
postpartum		
*p*-value	0.000	0.000

^1^*EPDS* refers to Edinburgh Postnatal Depressive Scale

Women with EPDS scores of 13 or above in mid-pregnancy were more likely to score high on Factor 1, compared to women without depressive symptoms (p 0.002). Depressive symptoms after birth (EPDS > 12) were associated with higher scores on both Factor 1 and Factor 2, suggesting that women with depressive symptoms after birth are more likely to present with impaired bonding and negative feelings towards the baby. The most impaired bonding scores, however, were seen in the composite variable where women with depressive symptoms both during pregnancy and at postpartum presented with the highest mean scores in Factor 1, and together with women who had depressive symptoms at only postpartum, in Factor 2.

## Discussion

While previous studies have primarily focused on fear of birth during pregnancy and depressive symptoms, the present study adds to the literature by including prenatal attachment and postpartum bonding. The main finding of this study was the association between prenatal attachment and postpartum bonding, where low prenatal attachment also showed impaired bonding. In addition, a strong association was found between depressive symptoms and impaired bonding in women with fear of birth. The principal component analysis failed to replicate earlier versions of the Postpartum Bonding Questionnaire.

### Low prenatal attachment and impaired bonding

The instruments included in the study, PAI [34], EPDS [302] and PBQ [18] showed some degrees of association. First, the two factors of PBQ were strongly correlated. This is not surprising, as Factor 1 represents caring activities and the woman’s perceptions of motherhood, and Factor 2 comprised negative feelings towards the baby. It is likely that negative feelings would make a woman less joyful about motherhood and, therefore, less likely to plan for future caring activities. Another explanation, found in the association between higher scores on EPDS and higher scores on PBQ indicates that a woman identified with depressive symptoms might not perceive motherhood with enjoyment.

Previous studies have also shown that mental illness, stress, and lack of support are closely related to prenatal depression [40], and a depressed mother can have difficulties in developing prenatal attachment [41].

In the present study with a sample of women with fear of birth we found associations between low prenatal attachment, high levels of depressive symptoms and impaired bonding. Similar findings have been reported in an Italian study showed impaired bonding three months after birth in women with fear of birth after controlling for depressive symptoms. The authors concluded that fear of birth itself is a factor for which directed interventions during pregnancy are needed [24]. It was suggested that depressive symptoms could be identified during pregnancy, either through a screening procedure or by the woman self-reporting if she perceives a trustful relationship with her midwife. 

There was also an association between the factors of PBQ and the subscales of PAI, with one exception, which was between Factor 2 in PBQ and the subscale *differentiation.* This particular subscale refers to perceptions of the baby’s personality and activities. The other subscales, *anticipation* and *interaction,* were correlated to both factors on the PBQ. Previous studies have shown that mothers with positive prenatal attachment also have more physical contact with the baby [42, 43].

In clinical practice, it might be important to strongly focus on interventions to increase prenatal attachment and thereby contribute to the bonding process. Midwives have a unique opportunity, during the regular antenatal visits, to help women interact with their unborn babies. Previous studies have suggested that during abdominal palpations women might be encouraged and instructed to use a hands-on approach to sense the position of the baby and the different body parts, which results in a higher maternal awareness of the baby [16, 44]. Midwives need to be compassionate and go beyond simply providing information and the use of checklists, which might diminish the signals and communication about the transition to motherhood [45].

### Background characteristics and postpartum bonding

The result of the present study not only showed associations between the instruments used, but also identified certain background characteristics that should be considered in relation to the factors of the postnatal bonding. First we determined that women who were not living with a partner in mid-pregnancy were more likely to report impaired bonding two months after birth. We do not know if their civil status changed over time, but it is likely that an unstable relationship could involve low social support, which are findings also shown in a Danish prospective cohort study [2]. In addition, an Italian study conducted on first-time mothers showed that women with fear of birth also reported lower scores in couple adjustment, with increased levels of anxiety and fear of birth [46].

Secondly, we found that, in this sample of women with fear of birth, that primiparous women were more likely to present with impaired bonding. This finding contradicts the results from studies that reported that multiparous women were more at risk of attachment [16] and bonding problems [2], and the latter review suggested that when a woman becomes a mother for the first time, she may have more time and attention for her pregnancy and child, which may positively affect mother-to-infant bonding. We do not know the reason for primiparous women’s higher scores on the PBQ, but one possible explanation could be that women identified with fear of birth might suffer from other traumas. Another explanation is that the women were preoccupied with their fear of birth during pregnancy and had not sufficiently processed the prenatal attachment [9]. Yet another explanation could be that they had not received any help with their emotional well-being, e.g. depressive symptoms.

The most impaired bonding was seen in the composite variable where women with depressive symptoms both during pregnancy and postpartum presented with the highest mean scores in Factor 1, and together with those who had depressive symptoms only after birth, in Factor 2. Having depression during a period that is usually associated with joy and happiness, probably creates long periods with increased levels of stress. Most pregnant women in Sweden are fully aware of the options of treatment for fear of birth, mainly counselling that is offered in all Swedish hospitals [47]. Screening for fear of birth is increasing in antenatal care in Sweden [48], but screening for depressive symptoms needs to be further developed/introduced during pregnancy. The increase in young women’s mental ill-health has been acknowledged, but might still arouse feelings of shame, during a period when you are supposed to be happy, as shown by Nagle and Farrelly, 2018 in an Irish interview study [49]. Evaluation of women’s mental health could also be challenging for midwives if treatment options and referrals are lacking.

Finally, this study adds to the growing body of research focusing on the link between prenatal attachment and postnatal bonding. We can only agree with the conclusions based on the review of 19 studies [9], that a regular screening process of fear of birth, depressive symptoms and prenatal attachment may be the first part of a preventive program that could have an impact on the development of bonding after birth.

### Methodological considerations

This study design is compromised by conducting a secondary analysis with a mix of women from the treatment groups. The self-reported questionnaires, the lack of psychiatric clinical diagnoses to verify depression and the under-representation of foreign-born women in the study sample reduce its capacity to be generalised to wider populations of pregnant women. The fairly small sample size and its specific characteristics, i.e., fear of birth, further limits the generalisability. Another limitation could be lack of control group as all women presented with fear of birth.

The strength of the study lies in its longitudinal design and the use of validated scales to measure depressive symptoms, fear of birth, prenatal attachment and postpartum bonding. The Principal Component Analysis failed to replicate previous studies that used the Postpartum Bonding Questionnaire. We chose to use the 16-item scale as it seemed most relevant in a Swedish context. We believe that the 25-item scale includes questions that would have yielded less variability, such as questions about abuse of the baby. In Sweden, parents are by law forbidden to act violently on children. Hairstone et al. [37] also excluded these questions due to ethical reasons.

## Conclusion

A focus on women’s mental health during pregnancy is necessary in order to avoid negative effects of impaired bonding on the infant. Depressive symptoms could be concurrent with fear of birth and, therefore, it is important to use screening procedures for both of these during pregnancy. Caregivers who meet women during pregnancy need to acknowledge prenatal attachment and thereby influence adaptation to motherhood.

## Data Availability

The datasets used and/or analysed during the current study are available from the corresponding author on reasonable request.

## Abbreviations

PBQ: Postpartum Bonding Questionnaire
PAI: Prenatal Attachment Inventory
EPDS: Edinburg Postnatal Depression Scale
FOBS: Fear Of Birth Scale
